# Noninvasive and invasive evaluation of cardiac dysfunction in experimental diabetes in rodents

**DOI:** 10.1186/1475-2840-6-14

**Published:** 2007-04-26

**Authors:** Rogério Wichi, Christiane Malfitano, Kaleizu Rosa, Silvia B De Souza, Vera Salemi, Cristiano Mostarda, Kátia De Angelis, Maria Claudia Irigoyen

**Affiliations:** 1 Hypertension Unit, Heart Institute (INCOR), University of São Paulo, Medical School, São Paulo, Brazil; 2 São Judas Tadeu University, Human Movement Laboratory, São Paulo, Brazil; 3 Nephrology Department, Federal University of São Paulo, São Paulo, Brazil; 4 Experimental Pathophysiology Program, University of São Paulo, Medical School, São Paulo, Brazil; 5 Cardiomyopathies Unit, Heart Institute (INCOR), University of São Paulo, Medical School, São Paulo, Brazil

## Abstract

**Background:**

Because cardiomyopathy is the leading cause of death in diabetic patients, the determination of myocardial function in diabetes mellitus is essential. In the present study, we provide an integrated approach, using noninvasive echocardiography and invasive hemodynamics to assess early changes in myocardial function of diabetic rats.

**Methods:**

Diabetes was induced by streptozotocin injection (STZ, 50 mg/kg). After 30 days, echocardiography (noninvasive) at rest and invasive left ventricular (LV) cannulation at rest, during and after volume overload, were performed in diabetic (D, N = 7) and control rats (C, N = 7). The Student t test was performed to compare metabolic and echocardiographic differences between groups at 30 days. ANOVA was used to compare LV invasive measurements, followed by the Student-Newman-Keuls test. Differences were considered significant at P < 0.05 for all tests.

**Results:**

Diabetes impaired LV systolic function expressed by reduced fractional shortening, ejection fraction, and velocity of circumferential fiber shortening compared with that in the control group. The diabetic LV diastolic dysfunction was evidenced by diminished E-waves and increased A-waves and isovolumic relaxation time. The myocardial performance index was greater in diabetic compared with control rats, indicating impairment in diastolic and systolic function. The LV systolic pressure was reduced and the LV end-diastolic pressure was increased at rest in diabetic rats. The volume overload increased LVEDP in both groups, while LVEDP remained increased after volume overload only in diabetic rats.

**Conclusion:**

These results suggest that STZ-diabetes induces systolic and diastolic dysfunction at rest, and reduces the capacity for cardiac adjustment to volume overload. In addition, it was also demonstrated that rodent echocardiography can be a useful, clinically relevant tool for the study of initial diabetic cardiomyopathy manifestations in asymptomatic patients.

## Background

Diabetes is a chronic metabolic disorder associated with secondary complications in the cardiovascular system and autonomic control in humans and animals [1-4]. Diabetic cardiomyopathy, a myopathic state independent of macrovascular complications, is the leading cause of death in diabetic patients [2]. Diabetic cardiomyopathy was implicated when diabetic patients at preclinical stages were found to exhibit a shortened left ventricular ejection time, a longer pre-ejection period, and elevated end-diastolic pressure [5]. However, the time-course of diabetic cardiomyopathy is still controversial, because reports have shown myocardial dysfunction at different times in the disease process.

Different methodologies have been used to investigate systolic and diastolic function in diabetic patients and rats. Assessment of cardiac function in experimental diabetes has relied on ex vivo [3] or in vivo [6-8] techniques. The ex vivo technique requires sacrifice of the animals and is devoid of autonomic reflexes and ventricular-vascular coupling. Although LV cannulation is a well-established, precise invasive method, not devoid of autonomic reflexes and ventricular-vascular coupling, this technique is limited because of the difficulty in keeping a catheter in the left ventricle throughout the long study period, and it is not without risk and does not allow assessment of the time course of cardiovascular changes. On the other hand, echocardiography has been used in several studies to demonstrate the time course of cardiovascular changes for different pathologies [9-12]. It is well established that clinically apparent diabetic cardiomyopathy may take several years to develop, but echocardiography can detect significant abnormalities early at the onset of symptomatic heart failure. Although several studies have used echocardiography as one noninvasive methodology to identify cardiac dysfunction associated with diabetes mellitus, no previous studies have used one integrated approach, ie, echocardiography and in vivo hemodynamics, to evaluate cardiac function in an experimental diabetes model. So the purpose of the present study was characterization of early myocardial dysfunction performed at the same time by noninvasive echocardiography and invasive LV catheterization in STZ-induced diabetic rats.

## Methods

Male Wistar rats (230 – 260 g) were obtained from the animal facilities at the University of São Paulo Medical School, São Paulo, Brazil. The rats received standard laboratory chow and water ad libitum. They were housed in individual cages in a temperature-controlled room (22°C) with a 12-h dark-light cycle. All procedures and protocols used were in accordance with the Guidelines for Ethical Care of Experimental Animals and were approved by the International Animal Care and Use Committee.

### Experimental diabetic model

The rats were randomly assigned to 1 of 2 groups: control (C, N = 7), diabetic (D, N = 7). Animals were made diabetic by a single injection of STZ (50 mg/Kg, iv; Sigma Chemical Co., St. Louis, MO, USA) dissolved in citrate buffer, pH 4.5. Food was withheld from the rats for 6 hours before STZ injection. Control rats received a placebo (10 mM citrate buffer, pH 4,5) after a similar fasting period.

Twenty-four hour urine was collected 29 days after diabetes induction. Animals were placed in a metabolic cage and were allowed free access to food and water during the collection period. Urine was collected and centrifuged at 1,000 g for 10 minutes to remove particles, and the volume was recorded. Urine samples were stored at -20°C for biochemistry analysis (Cobas Integra 700-Roche, Swiss).

### Noninvasive evaluation of cardiac function

Echocardiographic indices were obtained according to the recommendations of the American Society of Echocardiography. Transthoracic echocardiography was performed in control and diabetic animals at 30 days, by double-blind observers with the use of a SEQUOIA 512 (ACUSON Corporation, Mountain View, CA), which offers a 10–13 MHz multifrequency linear transducer. Images were obtained with the transducer on each animal's shaved chest (lateral recumbence). To optimize the image, a transmission gel was used between the transducer and the animal's chest (General Imaging Gel, ATL. Reedsville, PA, USA). Animals were scanned from below, at a 2-cm depth with focus optimized at 1 cm. All measurements were based on the average of 3 consecutive cardiac cycles. Rats were anesthetized with a combination of ketamine hydrochloride 50 mg/kg and xylazine 10 mg/kg IP. Wall thickness and LV dimensions were obtained from a short-axis view at the level of the papillary muscles. LV mass was calculated by using the following formula, assuming a spherical LV geometry and validated in rats: LV mass = 1,047 × [(LVd+PWd+IVSd)3 - LVd3], where 1,047 is the specific gravity of muscle, LVd is LV end-diastolic diameter, PWd is end-diastolic posterior wall thickness and IVSd is end-diastolic interventricular septum thickness. In addition, another index of morphology was evaluated, the relative wall thickness (RWT), which is expressed by 2 × PWD/LVd. It represents the relation between the LV cavity in diastole and the LV posterior wall. LV fractional shortening was calculated as (LVd-LVs)/LVd × 100, where LVs is LV end-systolic diameter. Two-dimensional guided pulsed-wave Doppler recordings of LV inflow were obtained from the apical 4-chamber view. Maximal early diastolic peak velocity (E) and late peak velocity (A) were derived from mitral inflow. The LV outflow tract velocity was measured just below the aortic valve, from an apical 5-chamber view. The velocity of circumferential fiber shortening (VCF) was measured following the formula (LVd-LVs)/(LVd × ET), where ET is the ejection time. The sample volume was then placed between the mitral valve and LV outflow tract so that the aortic valve closure line and the onset of mitral flow could be clearly identified. Isovolumic relaxation time (IVRT) was taken from aortic valve closure to the onset of mitral flow. Global cardiac function was evaluated by using the Myocardial Performance Index (MPI), which is the ratio of total time spent in isovolumic activity (isovolumic contraction time and isovolumic relaxation time) to the ejection time (ET). These Doppler time intervals were measured from the mitral inflow and LV outflow time intervals. Interval "*a*", from the cessation to onset of mitral inflow, is equal to the sum of the isovolumic contraction time, ET and isovolumic relaxation time. Ejection time "*b*" is derived from the duration of the LV outflow Doppler velocity profile. The MPI was calculated with the formula (*a-b*)/*b*.

### Invasive evaluation of cardiac function

LV function was measured also invasively in anesthetized rats (pentobarbital sodium, 40 mg/Kg). One catheter of PE-50 was inserted into the right carotid artery and advanced into the LV, and a second catheter of PE-50 was inserted into the jugular vein for saline and drug administration. Ventricular pressure signals were measured with a transducer and conditioner (Hewlett Packard 8805C, Waltham, MA) and digitally recorded (5 min) with a data acquisition system (WinDaq, 2-kHz, DATAQ, Springfield, OH). The recorded data were analyzed on a beat-to-beat basis to quantify changes in LV pressure. The following indices were obtained: heart rate (HR), LV systolic pressure (LVSP), LV end-diastolic pressure (LVEDP), and maximum rate of LV pressure rise and fall (+dP/dt max and -dP/dt max). After LV pressure basal records, the LV function was recorded during a volume overload protocol performed by an injection of saline (0.27 mL/min/kg) during 3 minutes adapted from Souza's study [13]. According to the volume overload protocol, the restoration of LV parameters was measured there for 3 minutes.

Data are reported as means ± SEM, and the Student *t *test was performed to compare metabolic and echocardiographic differences between different groups. Two-way ANOVA was used to compare LV invasive measurements, followed by the Student-Newman-Keuls test. Differences were considered significant at P < 0.05 for all tests.

## Results

Rats treated with STZ not only developed hyperglycemia (340 ± 7.6 vs. 87 ± 10 mg/dL, D vs. C, *P *< 0.0001) but also polydipsia (172 ± 6.5 vs. 40 ± 7 mL/24 h, D vs. C, P < 0.0001), polyuria (125 ± 4.4 vs. 9 ± 0.7 mL/24 h, D vs. C, P < 0.0001), glucosuria (12 ± 0.5 vs. 0.0026 ± 0.0003 mg/dL, D vs. C, P < 0.0001), proteinuria (0.02 ± 0.002 vs. 0.01 ± 0.0009 mg, D vs. C, P < 0.0001), and uremia (1.6 ± 0.09 vs. 0.27 ± 0.03 mg, D vs. C, P < 0.0001). Body weight was reduced in the diabetic group (270 ± 8 g) compared with weight in the control group (360 ± 11 g, P < 0.0001).

### Noninvasive evaluation of cardiac function

Echocardiography 30 days after STZ demonstrated that LV internal dimension (cm) during diastole increased (0.73 ± 0.03 vs. 0.63 ± 0.03, D vs. C, P < 0.05), and the thickness of the interventricular septum and of the posterior wall during diastole decreased (0.101 ± 0.003 vs. 0.138 ± 0.005 and 0.1 ± 0.003 vs. 0.138 ± 0.005, D vs. C, P < 0.001, respectively) in diabetic rats in relation to control rats. RWT was significantly lower in diabetic (0.27 ± 0.016) than in control rats (0.40 ± 0.01, P < 0.05). LV systolic function, as expressed by fractional shortening (%) (34 ± 3.7 vs. 39 ± 3.7, D vs. C, P < 0.05), ejection fraction (%) (69 ± 0.02 vs. 75.4 ± 0.015, D vs. C, P < 0.05) and VCF (circ/sec) (0.003 ± 0.0002 vs. 0.004 ± 0.0003, D vs. C, P < 0.01) was reduced in diabetic group compared with that in the control group. LV diastolic function was observed after 30 days of STZ-induced diabetes, as expressed by reduced E-wave (m/s) (0.44 ± 0.036 vs 0.53 ± 0.04, D vs. C, P < 0.05) and increased A-wave (m/s) (0.4 ± 0.04 vs. 0.33 ± 0.02, D vs. C, P < 0.05) in diabetic animals when compared with control animals. The diabetic group had a reduced E/A ratio (1.33 ± 0.09) compared with the control group (1.62 ± 0.07, P < 0.05). Deceleration time of E-wave (EDT, ms) (39 ± 1.3 vs. 30 ± 1.7, D vs. C, P < 0.0001) and IVRT (ms) (40.6 ± 1.65 vs. 31.6 ± 1.5, D vs. C, P < 0.05) were increased in the diabetic group compared with the control group, reflecting early diastolic dysfunction by slow relaxation. Statistical comparison of normalized EDT and IVRT by HR during echocardiography corroborated differences between groups observed using their absolute values. MPI was greater in the diabetic group (0.41 ± 0.014) compared with that in the control group (0.29 ± 0.054, P < 0.0001).

### Invasive evaluation of cardiac function

**Figure 1:**
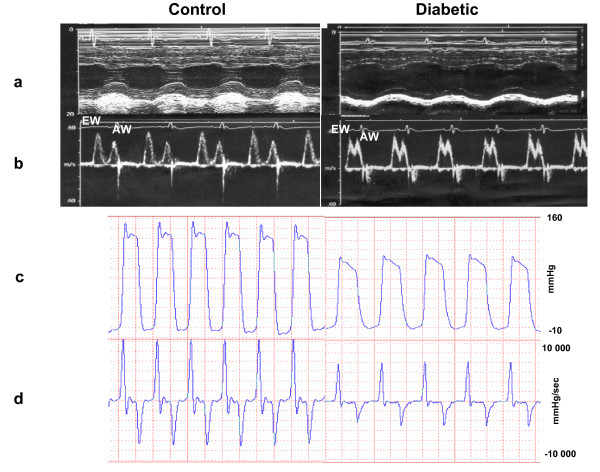
Left ventricle cavity (a), E-wave (EW, b), A-wave (AW, b), original record of basal left ventricle pressure (c), and +dP/dt max and -dP/dt max of left ventricle pressure (d) in control and diabetic rats.

As can be seen in Table 1, invasive measurements of cardiac function by LV cannulation demonstrate systolic and diastolic impairment at rest and during and after a volume overload protocol in diabetic rats compared with that in control rats. Figure 1 shows an original basal record of LV pressure and +dP/dt max and -dP/dt max of LV pressure in a control rat and a diabetic rat. The diabetic group had a reduction in LVSP (17%) and in HR (14%) in relation to the control group at baseline. The LVEDP, an index of congestive heart failure, showed a significant increase (57%) in diabetic rats compared with that in controls at baseline. Resting maximum rates of rise (+dP/dt max) and fall (-dP/dt max) in LV pressure were also impaired after diabetes.

**Table 1:** Left ventricular function at baseline, during, and after volume overload in the control and diabetic groups.

**Parameter**	**Group (n = 7)**	**BASELINE**	**VOLUME OVERLOAD**			**POST VOLUME OVERLOAD**		
			
			**1 min**	**2 min**	**3 min**	**1 min**	**2 min**	**3 min**
**LVSP**	**C**	136 ± 4.7	125 ± 6.5	135 ± 7	134 ± 6	139 ± 7.8	134 ± 8.8	138 ± 6
**(mm Hg)**	**D**	114 ± 6*	116 ± 5.8*	112 ± 5 *	114 ± 6 *	107 ± 10 *	102 ± 4 *	107 ± 10*
								
**LVEDP**	**C**	4.6 ± 0.3	7.8 ± 0.6	8.0 ± 0.6	9.1 ± 0.56	11 ± 1.3	9 ± 1.1	9.4 ± 1.1
**(mm Hg)**	**D**	7.4 ± 0.2*	9.9 ± 0.5*#	12.3 ± 0.55*#	11.5 ± 0.72*#	17 ± 1.3 *#†	16 ± 0.7 *#†	15 ± 1.1 *#†
								
**HR**	**C**	350 ± 14	327 ± 10	333 ± 11.5	340 ± 7.5	352 ± 5	360 ± 6	360 ± 6
**(beats/min)**	**D**	307 ± 8.5*	300 ± 6 *	300 ± 7*	302 ± 7*	297 ± 1.5*	298 ± 2*	294 ± 3.5 *
								
**+dP/dt max**	**C**	9229 ± 463	8052 ± 741	8699 ± 653	8595 ± 457	8858 ± 504	9273 ± 569	9264 ± 603
**(mm Hg/sec)**	**D**	6566 ± 609 *	5861 ± 584 *	5760 ± 770 *	6087 ± 1017*	5320 ± 1287 *	5844 ± 1217*	5712 ± 1247*
								
**-dP/dt max**	**C**	-6845 ± 379	-6446 ± 576	-6543 ± 600	-6139 ± 514	-6672 ± 646	-6320 ± 858	-6272 ± 707
**(mm Hg/sec)**	**D**	-4746 ± 589*	-4804 ± 580*	-4678 ± 580*	-4152 ± 694*	-3706 ± 614 *	-3557 ± 561*	-3246 ± 653*

Data are reported as means ± SEM; LVSP: left ventricle systolic pressure; HR: heart rate; LVEDP: left ventricle end diastolic pressure; +dP/dt max: maximum rate of left ventricle pressure rise; -dP/dt max: maximum rate of left ventricle pressure fall; C: control group; D: diabetic group. * P ≤ 0.05 vs. controls; #P < 0.05 vs. baseline values; † P < 0.05 vs. volume overload values (2-way ANOVA).

During and after volume overload, the differences in LV function between groups remained the same as in the baseline period. However, the LVEDP increased in both groups during and after volume overload compared with their respective baseline values. Only diabetic rats showed an increase in LVEDP values in the post volume overload period in relation to LVEDP values during the volume overload period (Table 1).

## Discussion

The present study provides an integrated approach to the assessment of diabetic cardiac function in rats, using both noninvasive echocardiography and invasive hemodynamic evaluation; it provides important new insights into the pathophysiology of diabetic cardiomyopathy. The present data confirm our preliminary findings that STZ diabetes induces hyperglycemia, weight loss, and bradycardia. However, the major finding of this study is that rats with 30 days of STZ-induced-diabetes had impairment in cardiac structure and function as evidenced noninvasively by echocardiography and invasively by LV catheterization, and also that this LV dysfunction was exacerbated by volume overload.

### Noninvasive evaluation of cardiac function

Echocardiographic measurements of cardiac function and structure in the present experiments demonstrated both LV systolic and diastolic dysfunction in 30-day diabetic rats. In fact, several investigators have shown abnormalities in LV systolic function, diastolic function, and complaints in humans [10-12] and rats [9,14-16], by using a noninvasive method of evaluation. Joffe et al [7] observed diabetic cardiomyopathy, characterized by LV systolic and diastolic dysfunction, after two and a half months of STZ in rats. Akula et al [9] in a recent study used echocardiography to examine LV function in STZ rats over a definite course of time (2, 4, 8, and 12 weeks). They concluded that LV systolic and diastolic dysfunction was fully visible at 12 weeks of diabetes by this method, and that echocardiography is useful in diagnosing cardiac abnormalities in diabetic rats without the need for invasive histopathological procedures. In contrast with these authors, we observed systolic and diastolic dysfunction after 30 days of STZ-induced diabetes documented by reduced ejection fraction, fractional shortening, and E-wave and increased A-wave and EDT, as well as by reduced VCF and increased IVRT observed in diabetic animals compared with controls. Corroborating our data, Yu et al [17] observed significant deficits in myocardial morphology and functionality by using magnetic resonance imaging at 4 weeks of diabetes in STZ-induced diabetic mice. In another study, Mihm et al [14] showed reductions in HR and EDT after 3 days of STZ-induced diabetes, and these reductions progressed throughout the 56-day period. Furthermore, these authors observed that systolic dysfunction was detected only after 35 days of study.

LV cavity dilatation accompanied by no changes in wall thickness has been observed previously after 35 and 75 days [7,14] and 12 weeks [9] of diabetes. Joffe et al [7] demonstrated not only LV cavity dilatation, but also increased LV mass after 75 days of STZ induction in rats, suggesting evidence of cardiomyopathy characterized by eccentric hypertrophy. In contrast, in the present study, we observed LV cavity dilatation accompanied by decreased thickness of the LV posterior wall and interventricular septum after 30 days of diabetes, suggesting a reduction in LV mass. Similarly, Dobrzynki et al [18] observed reduced heart weight and LV mass associated with cardiac and renal function damage after 21 days of STZ-induced diabetes in rats. In addition, our study is the first to describe the RWT reduction in diabetic rats, indicating a probable reduction in LV mass, as previously demonstrated [19].

MPI is a simple and nongeometric index that combines systolic and diastolic functions independently of heart rate, not requiring frequency based normalization [20]. The importance of MPI in this study is that it is an HR-independent measure unlike transmitral flow velocities. MPI also has not been used previously to investigate cardiomyopathy in diabetic rats. In our evaluations, MPI was increased in diabetic rats, suggesting global cardiac dysfunction in these animals. This result appears to correlate well with invasive measurements of systolic and diastolic measurements in humans [20] and animals [21]. Recently, MPI was validated in mice, and is strongly correlated with invasive measurements of the LV dP/dt max [21]. MPI has a prognostic value after myocardial infarction [22], as well as in adults with amyloidosis [23] and dilated cardiomyopathy [24], and is not affected by mitral regurgitation [25].

### Invasive evaluation of cardiac function

Dent et al [16] demonstrated that differences in diastolic function might be noninvasively quantified in diabetic hearts; however, these authors recognized that the lack of in vivo hemodynamic data was one limitation of their study. In our experiments, direct measurements of cardiac function corroborate diastolic and systolic LV function impairment observed after 30-day-induced diabetes by the echocardiographic approach. In vivo LV function evidenced reduced LVSP and +dP/dt max, reflecting a systolic dysfunction, increased LVEDP and attenuated -dP/dt max, showing diastolic dysfunction. These data associated with the impairment in MPI reported in the diabetic group in the present study corroborate the positive correlation between these ventricular indexes, as previously shown in mice [21]. In fact, similar alterations in LVEDP, contractility, bradycardia, reduced cardiac output, and renal damage were evidenced after 21 days of STZ in rats. Reduction in HR in diabetic rats has been attributed to changes in sinoatrial node [1,3,26], although functional alterations in the cholinergic mechanism cannot be excluded as a causal factor. Joffe et al [7] showed the in vivo reduced peak of LV systolic pressure and increased LVEDP, as well as in vitro attenuation of LV +dP/dt max and -dP/dt max after 75 days of STZ-induced diabetes in rats. Another study in which in vivo LV cannulation was performed showed a decrease in LV +dP/dt max and -dP/dt max after 15 days of STZ injection in rats [8]. Impairment in cardiovascular function in mice was also observed in diabetic-infarcted mice at the same time point [27]. In a previous study by our group, we reported that the isolated hearts of 11-week STZ-induced diabetic rats did not have differences in LV isovolumetric systolic pressure, but had reduced contractility compared with isolated hearts in control rats [3].

In addition, our results show impairment in cardiac responses during and after volume overload in STZ-induced rats. A similar increase in LVEDP observed in control rats in the present experiments was previously demonstrated in the early stages of volume overload induced by A-V fistula in control rats [28]. However, in contrast with that observed in control rats, the high values of recorded LVEDP in STZ-induced rats remained elevated after volume overload, which may be explained by cardiac changes produced by STZ. Despite the fact that LV cavity dilatation allows major blood compliance, the reduced fractional shortening, ejection fraction, and VCF observed in STZ-induced rats are probably associated with the increase in LVEDP during and after volume overload, because the volume of blood can not be completely ejected.

Studies that have examined both systolic and diastolic dysfunction in diabetes suggest that the latter is more susceptible to preclinical changes. Diastolic dysfunction is not just a defect in active relaxation, but also in passive stiffness of the left ventricle [29]. The echocardiographic evidence of subclinical contractile dysfunction and diastolic filling abnormalities are predictive of subsequent chronic heart failure [30]. Patients with diastolic heart failure have an increased mortality of 5–8% compared with the control group [31]. Systolic dysfunction occurs late, often when patients have already developed significant diastolic dysfunction. However, the prognosis in patients with systolic dysfunction is an annual mortality of 15–20%, greater than mortality in patients with diastolic dysfunction [31]. Thus, the early detection of diastolic and systolic dysfunction can prevent worsening of this condition.

## Conclusion

Using the STZ model of diabetes, we have demonstrated that rodent echocardiography can be useful, because sensitive changes in systolic and diastolic performance were detected and markedly confirmed by in vivo direct LV measurements. Changes reported in the present experiments in cardiac performance are highly predictive of clinical findings, and further illustrate the value of this model of diabetic cardiomyopathy.

## Abbreviations

LV: left ventricle

STZ: streptozotocin

AP: arterial pressure

HR: heart rate

LVd: left ventricle end-diastolic diameter

LVs: left ventricle end-systolic diameter

PWd: end-diastolic posterior wall thickness

IVSd: end-diastolic interventricular septum thickness

RWT: relative wall thickness

E: maximal early diastolic peak velocity

A: late peak velocity

VCF: velocity of circumferential fiber shortening

ET: ejection time

IVRT: isovolumic relaxation time

MPI: myocardial performance index

LVSP: left ventricular systolic pressure

LVEDP: left ventricular end-diastolic pressure

+dP/dt max: maximum positive values of first derivative of left ventricular pressure over time

-dP/dt max: maximum negative values of first derivative of left ventricular pressure over time

EDT: deceleration time of E-wave

## Competing interests

The author(s) declare that they have no competing interests.

## Authors' contributions

All authors have equally contributed to the conception and drafting of the manuscript.
